# Occupational risk of COVID-19 related hospital admission in Denmark 2020–2021: a follow-up study

**DOI:** 10.5271/sjweh.4063

**Published:** 2022-12-30

**Authors:** Jens Peter Ellekilde Bonde, Lea Sell, Esben Meulengracht Flachs, David Coggon, Maria Albin, Karen Marieke Oude Hengel, Henrik Kolstad, Ingrid Sivesind Mehlum, Vivi Schlünssen, Svetlana Solovieva, Kjell Torén, Kristina Jakobsson, Christel Nielsen, Kerstin Nilsson, Lars Rylander, Kajsa Ugelvig Petersen, Sandra Søgaard Tøttenborg

**Affiliations:** 1 Department of Occupational and Environmental Medicine, Bispebjerg and Frederiksberg University Hospital, Copenhagen, Denmark; 2 Department of Public Health, University of Copenhagen, Copenhagen, Denmark; 3 MRC Lifecourse Epidemiology Centre, University of Southampton, Southampton, UK; 4 Unit of Occupational Medicine, Institute of Environmental Medicine, Karolinska Institutet, Stockholm, Sweden; 5 Centre for occupational and Environmental Medicine, Region Stockholm, Stockholm, Sweden; 6 Netherlands Organization for Applied Scientific Research TNO, Department of Work Health Technology, Leiden, The Netherlands; 7 Department of Occupational Medicine, Danish Ramazzini Centre, Aarhus University Hospital, Århus, Denmark; 8 National Institute of Occupational Health (STAMI), Oslo, Norway; 9 Institute of Health and Society, University of Oslo, Oslo, Norway; 10 Department of Public Health, Danish Ramazzini Centre, Aarhus University, Aarhus, Denmark; 11 National Research Center for the Working Environment, Copenhagen, Denmark; 12 Finnish Institute of Occupational Health, Helsinki, Finland; 13 Occupational and Environmental Medicine, Sahlgrenska University Hospital, Gothenburg, Sweden; 14 Discipline of Occupational and Environmental Health, University of KwaZulu-Natal, Durban, South Africa; 15 School of Public Health and Community Medicine, Sahlgrenska Academy, University of Gothenburg, Sweden; 16 Division of Occupational and Environmental Medicine, Department of Laboratory Medicine, Lund University, Lund, Sweden; 17 Division of Public Health, Kristianstad University, Kristianstad, Sweden

**Keywords:** cohort study, epidemiology, healthcare, industry, ISCO-08, job, NACE, pandemic, SARS-CoV-2

## Abstract

**Objective:**

Mounting evidence indicates increased risk of COVID-19 among healthcare personnel, but the evidence on risks in other occupations is limited. In this study, we quantify the occupational risk of COVID-19-related hospital admission in Denmark during 2020–2021.

**Methods:**

The source population included 2.4 million employees age 20–69 years. All information was retrieved from public registers. The risk of COVID-19 related hospital admission was examined in 155 occupations with at least 2000 employees (at-risk, N=1 620 231) referenced to a group of mainly office workers defined by a COVID-19 job exposure matrix (N=369 341). Incidence rate ratios (IRR) were computed by Poisson regression.

**Results:**

During 186 million person-weeks of follow-up, we observed 2944 COVID-19 related hospital admissions in at-risk occupations and 559 in referents. Adjusted risk of such admission was elevated in several occupations within healthcare (including health care assistants, nurses, medical practitioners and laboratory technicians but not physiotherapists or midwives), social care (daycare assistants for children aged 4–7, and nursing aides in institutions and private homes, but not family daycare workers) and transportation (bus drivers, but not lorry drivers). Most IRR in these at-risk occupations were in the range of 1.5–3. Employees in education, retail sales and various service occupations seemed not to be at risk.

**Conclusion:**

Employees in several occupations within and outside healthcare are at substantially increased risk of COVID-19. There is a need to revisit safety measures and precautions to mitigate viral transmission in the workplace during the current and forthcoming pandemics.

The COVID-19 pandemic caused by SARS-CoV-2 virus posed a new and potentially fatal occupational hazard. Transmission of SARS-CoV-2 infection could be expected not only within households, and through private and public social contacts in leisure time, but also in the workplace – and particularly in jobs (equivalent to ‘occupations’ throughout) that entail frequent close contact with other people or selective contact with infected individuals (1–3). Transmission in the workplace would both lead to potentially lethal occupational disease among employees and accelerate the spread of infection across society (2). Understanding of the risks of COVID-19 by occupation is important from both perspectives. It is needed to both prioritize public health interventions aimed at containing the spread of the epidemic and optimize protection of workers’ health. In addition, it is important to inform decisions on compensation for COVID-19 as an occupational disease.

Ideally, research into risks of COVID-19 by occupation would be based on cases ascertained through systematic prospective follow-up of large cohorts of workers, with regular ascertainment of symptoms, clinical signs, and polymerase chain reaction (PCR) testing. As that has not been feasible, researchers have been forced to use proxy measures of incidence based on: PCR testing (4–11), measurements of specific immunoglobulins against SARS-CoV-2 (12–14), symptoms and sickness absence (6), COVID-19 related hospital admission (7, 15, 16), or mortality from the disease (7, 15, 17–22). In most studies to date, with broad coverage of occupations, mortality from COVID-19 has been the outcome measure (7, 15, 17–22).

While the majority of studies so far have focused on healthcare workers (1, 5–9, 12–14, 21, 23–29), a number of studies in the first year of the pandemic have indicated that risk may also be increased in other occupations (16, 18–20, 22). For instance, an English mortality study, which included about 2 500 COVID-19 related deaths, found that taxi and bus drivers, shop assistants, and social workers had up to four-fold increases in age-adjusted mortality (19), and a Swedish national study also observed increased mortality in taxi and bus drivers (18). A Norwegian study found increased risk of COVID-19 related hospitalization in dentists, but numbers were small (4). A Swedish study found substantially increased risk of COVID-19 hospitalization among employees, who according to a job exposure matrix (JEM) worked in close proximity to other people or had contact with infected patients (16).

As yet, however, few studies have quantified differences in risk of severe disease across highly specific occupations. Among research priorities outlined in an editorial, identification of specific occupations at high risk of COVID-19 was considered of high value as preparation for future pandemics (30). The attention to precautions and protective equipment among healthcare professionals is probably high compared to other occupations, where the awareness of risk may be far less, and more training and protection needed. According to a newly developed expert-rated JEM (31), 87% of the total of 423 job groups of the International Standard Classification of Occupations (ISCO-08) may carry a higher risk of SARS-CoV-2 infection. This accounts for 85% of Danish employees, of whom <15% are working in the healthcare sector.

The aim of the current study was to systematically quantify the risk of COVID-19-related hospital admission among employees across all occupations with >2000 employees during the first two years of the pandemic. Hospital admission was considered a proxy for severe COVID-19. The study took advantage of access to updated information in a large nationwide cohort. We focused on hospital admission as an outcome to achieve greater statistical power than an analysis of mortality, and to avoid the possibility of bias from differential access to testing by occupation (32).

## Methods

### Population

The study cohort comprised all Danish employees aged 20–69 years at 1 January 2020 (N=2 451 542). It was identified from records in the Work Classification Module at Statistics Denmark, as a subset of the Danish Occupational Cohort with eXposure data (DOC*X) (33). Other persons from the same households as cohort members (N=1 503 892, 74% ≤20 years of age; 1.4% ≥70 years of age) were identified through the residential address in order to account for viral transmission in the family. Permissions to remotely retrieve, compile and analyze pseudonymized data at a secured and logged platform at Statistics Denmark were obtained from the Danish Data Protection Agency (P-2020-897), Statistics Denmark (P-708121) and the National Board of Health Data (FSEID-00005368). This process started in August 2020, and access to the data-files was provided in December 2021.

### Occupation and industry

Occupation and industry codes are primarily provided by employing companies but other sources of information such as tax records, trade union membership, and educational records are also used by Statistic Denmark. This allowed assignment of job titles according to the Danish version of the International Standard Classification of Occupations (DISCO-08) (34), for 86% at the 4-digit level, and assignment of industry codes according to the Danish version of the Statistical Classification of Economic Activities in the European Communities (DB07) (35) for 100% at the 2-digit level. When we retrieved data for this study in December 2021, the most updated information on occupational and economic activity codes was from December 2019. Therefore, we did not have data on changes of occupation in 2020 and 2021. With few exceptions, the Danish classifications are identical to the corresponding international classifications (ISCO-08 and NACE Rev. 2).

With one exception, it was not possible to infer missing DISCO-08 codes from known DB07 codes because individual DB07 codes are associated with a wide miscellany of DISCO-08 codes. However, as 75% of employees within ‘hairdressing and other beauty treatment’ (DB07 code 96.02.10) were hairdressers (DISCO-08 code 5141), all individuals with a missing DISCO-08 code within this industry (N=1135) were assigned the 5141 DISCO-08 code.

### Outcome ascertainment

Data on admission to hospital for COVID-19-related disease from 1 January 2020 through 14 December 2021 were obtained from a daily updated database with national coverage kept at Statens Serum Institut, Copenhagen. A hospital admission was defined as COVID-19 related if a SARS-CoV-2 PCR swab test was positive up to 14 days prior to admission, and if the hospital stay was >12 hours (an algorithm used by Statens Serum Institut). An examination of ICD-10 diagnoses available from the National Patient Registry indicated that about 2.5% of cases using this definition were probably attributable to other causes such as psychiatric, traumatic, or obstetric disorders.

### Reference group

The reference group was defined *a priori* as occupations classified to the lowest level of potential occupational exposure to SARS-CoV-2 by an expert-rated COVID-19 JEM (31). Eight domains addressing four factors on transmission risk (type, number, proximity, and location of social contacts at work), two mitigation measures (social distancing and face covering) and two job insecurity factors (income insecurity and proportion of migrants) were each rated on a scale from 0 (low risk of exposure) to 3 (high risk of exposure). The reference group was defined by the 50 job codes (4-digit DISCO-08 level) with low risk on each of the eight dimensions (sumscore_1-8_ = 0, N=369 341, 15% of the study cohort). The three largest job groups in the reference population were general office clerks, commercial sales representatives, and accounting associate professionals (41% of the reference group, DISCO-08 codes 4110, 3322 and 3313, respectively).

An overview of the subsets of the study population is displayed in table 1.

**Table 1:** Overview of the study population (numbers). COVID-19 risk estimates are provided for employees in the subgroups **indicated with bold**. [JEM=job exposure matrix.]

	Employees	4-digit DISCO-08 occupations	Incident COVID-19 cases
At-risk ^a^ occupations with >2000 employees	**1 620 231 ^b^**	155	2944
Industrial sector (DB07) with average 4-digit-DISCO-08 JEM sumscore <12–24	853 599	155	1751
JEM sumscore 1–12	126 575	97	183
JEM sumscore >12–24	**727 024 ^c^**	58	1568
Industrial sector (DB07) with average 4-digit-DISCO-08 JEM sumscore 1–12	766 632	155	1193
At-risk ^a^ occupations with ≤2000 employees	**124 287**	218	220
Referent occupations ^d^	369 341	50	559
Missing 4-digit DISCO-08 codes	**337 306**		693
Dead before start of follow-up week 8, 2020	377		0
Entire study population	2 451 542	423	4416

a Non-referent occupations.

b Occupational risk across all industrial sectors (displayed in table 3)

c Industrial sector-stratified risk estimates (displayed in table 2). Numbers in table 2 do not sum to corresponding numbers in table 1 because some occupations are present in several industrial sectors and some occupations are presented at a higher or lower DISCO-08 level.

d Low likelihood of occupational SARS-CoV-2 exposure according to an expert rated COVID-19 job exposure matrix with eight dimensions (sumscore = 0). Occupations that met this criterion were included in the reference group whatever their number of employees.

### Covariates

Using their unique personal identification numbers, we linked each cohort member with records in public registers hosted by Statistics Denmark to obtain information on a range of demographic, social and health variables at the end of 2019: sex, age, duration of education in years, country of origin, social position, household income, hospital admission for ≥1 of 11 chronic diseases during 2010–2019, geographical residential area and living in a municipality with a large city.

From data on household members, we defined variables indicating the size of the household sharing the same residence. The number of family members (excluding the index-person), who had ≥1 PCR swab test during the second or third week before any particular week during follow-up, and the results of those tests, were retrieved from the national testing database, which by mid December 2021 included results of about 40 million PCR tests. Tests were available free of charge throughout the country from early on in the first epidemic wave. The 2–3 week interval was specified to account for incubation period and disease development before hospitalization

Individual proxies of lifestyle factors in terms of sex-, age- and period-specific probability of current smoking and estimates of body mass index (kg/m^2^) were assigned by lifestyle JEM based on questionnaire information from several large random samples of the Danish population representative for 2010 (36).

From the DREAM register with longitudinal data on public benefit transfer payments to all Danish citizens (37), we obtained data on retirement each week during 2020 (data not available for 2021). Finally, we obtained information on the date of second vaccination against COVID-19 (if any) and vital status during 2020 and 2021.

### Statistical analysis

We examined incident hospital admission for COVID-19 related disease as a function of occupation and covariates. Crude and adjusted measures of association, in terms of incidence rate ratios (IRR) with 95% confidence intervals (CI), were computed by Poisson regression applied to counts of COVID-19 hospital admissions and weeks at-risk for all covariate cross tabulations. Follow-up started in week 8 of 2020 (the week before first hospital admissions for COVID-19 in Denmark) and continued until (and including) week 50 of 2021 (the last week with available data on COVID-19 admissions). Follow-up was censored at the first of: COVID-19 related hospital admission (N=4416); death (N=2615); emigration (N=18 112); retirement (N=33 427); or week 50 of 2021.

A power calculation based upon the outcome occurrence in the reference group indicated that a relative risk of 1.8 would be detectable in an occupation with 80% power at the 5% significance level if it included >2000 employees. Therefore, we report risk estimates for the non-referent occupations of this size or larger, while keeping all jobs regardless of size in the reference group. Occupations with ≤2000 employees and employees with missing DISCO-08 codes were analyzed as separate categories.

*Risk estimates by industrial sector*. To account for possible differences between the same occupation in different industrial sectors – for instance cleaners in hospitals versus cleaners in office buildings and nursing aides in institutions versus nursing aides in private homes – we present occupational risk stratified by industrial sectors with more likely exposure to SARS-CoV-2. These industrial sectors were selected by an average COVID-19 JEM sum score >12 (the median) for industries classified at the 2-digit DB07 level. DB07 groups were pooled when appropriate, considering type of industrial sector (for instance land-, sea and air transportation, DB07 codes 49, 50 and 51). In few instances we deviated from the 4-digit DISCO-08 classification by splitting the 4-digit groups into 6-digit groups to distinguish more specific occupations (for instance day care assistants by age of the children taken care of) or to pool 4-digit groups into 3-digit groups to obtain sufficient sample size – for instance generalist and specialized medical practitioners or waiters/bartenders (explicitly detailed in table 2). Separate risk estimates are reported for occupations with a COVID-19 JEM sum score >12 but for completeness a summary risk estimate for all occupations with COVID-19 sum scores ≤12 is also provided.

**Table 2:** Risk of Covid-19 related hospital admission in occupations with >2000 employees working in industrial sectors (2-digit DB07) and occupations (DISCO-08) with a COVID-19 job exposure matrix sumscore >12. Incidence rate ratios (IRR) with 95% confidence limits (CI) relative to employees in all occupations with unlikely occupational exposure to SARS-CoV-2 ^a^. Significant associations in **bold.**

Occupation (descending number employees)	DISCO-08 code	Employees (N)	COVID-19 admissions (N)	IRR (sex and age adjusted)	IRR fully adjusted ^b^	95% CI
HEALTHCARE (db07 code 86)						
Nursing Professionals	2221	51 156	104	1.65	**1.89**	**1.42–2.50**
Medical Practitioners	2211, 2212	18 518	44	1.64	**2.03**	**1.27–3.25**
Dental Assistants and Therapists	3251	8068	12	1.34	0.96	0.53–1.75
Healthcare Assistants	532120	7142	35	3.50	**3.54**	**2.34–5.36**
Physiotherapists	226410	6541	8	0.98	1.30	0.63–2.70
Medical Laboratory Technicians	3212	6229	16	2.06	**1.86**	**1.06–3.24**
Psychological Therapists	2634	5052	12	1.91	**2.52**	**1.37–4.65**
Cleaners and Helpers	9112	4045	15	2.59	1.53	0.87–2.69
Recreational Therapists	226910	3798	8	1.78	**2.35**	**1.10–5.02**
Dentists	226100	3351	6	1.26	1.23	0.54–2.80
Hospital Attendants (porters)	532130	3172	12	1.97	**1.94**	**1.06–3.56**
X-ray Technicians	3211	2438	7	2.22	**2.22**	**1.04–4.73**
Midwifery Professionals	2222	2042	<5	0.91	1.05	0.26–4.24
COVID-19 JEM sumscore 1-12		25 189	51	1.52	**1.61**	**1.20–2.17**
2000 employees		2864	10	1.91	2.08	1.08–3.98
Missing DISCO-08 code		11 762	18	1.12	1.47	0.65–3.33
SOCIAL CARE (DB07 code 87-88)						
Special Teaching Professionals	2357	31 529	50	1.10	1.10	0.78–1.55
Nursing Aides (institutions)	5321	27 224	62	1.66	**1.43**	**1.06–1.94**
Homecare Aides, private homes (DB07 8810)	532210	26 134	69	1.95	1.22	0.90–1.64
Nursing Aides (private homes)	532220	21 313	61	2.17	**1.67**	**1.21–2.29**
Teachers/Daycare Assistants. 0-3 years (DB07 889120)	531120	17 908	29	1.25	1.24	0.85–1.81
Teachers/Daycare Assistants.4-7 years (DB07 889130)	531120	11 150	29	1.83	**1.56**	**1.07–2.28**
Teachers/Daycare Assistants.7-15 years ((DB07 889140)	531120	4388	6	1.11	0.93	0.41–2.08
Nursing Professionals	2221	11 103	26	1.76	**1.81**	**1.18–2.77**
Family Daycare Workers	531110	9900	13	0.92	0.96	0.55–1.68
Social Work and Counselling Professionals	263500	4314	5	0.89	0.88	0.36–2.13
Cleaners	9112	4178	17	2.78	1.41	0.84–2.36
Primary School Teachers	2341	3316	5	1.11	1.05	0.43–2.54
Kitchen Helpers	9412	2353	<5	1.31	2.02	0.73–5.59
Physiotherapists	2226	2184	5	1.54	1.03	0.42–2.52
COVID-19 JEM sumscore 1-12		112 577	253	1.64	**1.28**	**1.07–1.52**
≤2000 employees		2195	<5	1.23	0.98	0.37–2.62
Missing DISCO-08 code		14 157	31	1.50	1.12	0.62–2.01
EDUCATION (DB07 code 85)						
Primary School Teachers	2341	72 524	113	1.09	1.16	0.93–1.45
University and Higher Education Teachers	2310	28 225	26	0.64	0.67	0.44–1.01
Secondary Education Teachers	233010	13 811	15	0.74	0.87	0.52–1.48
Early Childhood Educators	2343	12 487	22	1.25	1.21	0.78–1.86
Vocational Education Teachers	2320	10 074	13	0.74	0.77	0.44–1.34
Preschool Child Helpers	531120	5327	12	1.62	1.37	0.77–2.45
Education Managers	134500	4450	<5	0.50	0.57	0.21–1.54
Cleaners	9112	3704	14	2.40	1.60	0.89–2.87
COVID-19 JEM sumscore 1-12		27 185	60	1.40	1.39	1.04–1.85
≤2000 employees ^c^		6046	16	1.63	1.60	0.97–2.64
Missing DISCO-08 code		9248	20	1.42	1.29	0.65–2.57
TRANSPORTATION (DB07 code 49-51)						
Heavy Truck and Lorry Drivers	8332	14 351	22	0.78	0.84	0.52–1.36
Bus and Tram Drivers	8331	10 775	93	4.35	**2.46**	**1.79–3.40**
Car, Taxi and Van Drivers	8322	2858	18	3.37	1.60	0.94–2.70
Locomotive Engine Drivers	8311	2361	7	1.41	1.23	0.58–2.61
Travel Attendants and Travel Stuarts	5111	2090	<5	0.66	0.63	0.15–2.58
COVID-19 JEM sumscore 1-12		8312	15	1.03	0.84	0.50–1.43
≤2000 employees		5987	12	1.04	0.92	0.51–1.65
Missing DISCO-08 code		14 576	70	2.79	**1.49**	**1.12–1.97**
RETAIL SALES (DB07 code 47)						
Shop Sales Assistants, non-specialized (DB07 4711- 4719)	5223	17 734	22	1.03	0.94	0.59–1.50
Shop Sales Assistants, specialized (DB07 472-78)	5223	36 771	51	1.16	1.15	0.82–1.62
Cashiers and Ticket Clerks	5230	12 804	15	0.97	0.93	0.54–1.61
Retail Trade Managers	142010	5962	10	1.17	1.24	0.65–2.35
Service Station Attendants	5245	3592	<5	0.71	0.56	0.18–1.79
Pharmaceutical Technicians and Assistants	3213	3267	7	1.77	1.27	0.59–2.73
Shelf Fillers	9334	3248	9	1.96	1.41	0.71–2.80
Butchers and Fishmongers	7511	2973	<5	0.95	1.15	0.42–3.13
COVID-19 JEM sumscore 1-12		15 204	22	1.02	0.91	0.59–1.42
≤2000 employees		3984	12	2.20	**2.15**	**1.21–3.82**
Missing DISCO-08 code		20 760	32	1.12	0.94	0.65–1.35
ACCOMODATION, FOOD, BUILDING, PERSONAL AND PROTECTIVE SERVICES, RECREATION ACTIVITY (DB07 codes 55, 56, 81, 93, 96)						
Cleaners and Helpers (except private homes)	9112	27 849	107	2.82	1.20	0.89–1.61
Cooks	5120	10 496	9	0.67	0.50	0.25–0.99
Waiters and Bartenders	5131, 5132	10 332	9	0.84	0.67	0.34–1.33
Protective Service Workers	5411-5419	21 843	51	1.42	1.24	0.92–1.68
Kitchen Helpers	9412	7658	14	1.54	0.89	0.51–1.56
Building Caretakers	5153	5660	15	1.35	1.18	0.69–2.04
Hairdressers and Cosmetologists	514	5398	10	1.74	1.49	0.74–3.01
Fast Food Preparers	9411	3610	<5	0.48	0.36	0.09–1.47
Gardeners and Horticultural Growers	6113	2742	<5	0.23	0.23	0.03–1.64
Hotel Receptionists	4224	2679	<5	0.66	0.54	0.13–2.20
COVID-19 JEM sumscore 1-12		22 311	37	1.04	0.87	0.61–1.24
≤2000 employees ^c^		12 786	31	1.66	1.38	0.95–2.02
Missing DISCO-08 code		50 569	143	2.19	2.03	0.77–5.32
Reference (all occupations with unlikely occupational SARS-CoV-2 exposure) ^a^		369 341	559	1.00	1.00	

a Likelihood of occupational SARS-CoV-2 exposure according to a population-based international expert-rated job exposure matrix that assesses four measures of number of close indoor contacts at work, two mitigation measures and two job insecurity measures, each rated on a scale from low (0) to high (3),

b Adjustment for sex, age (10 year groups), duration of education at baseline (5 groups), number of hospital admissions for one or more of 11 chronic diseases in the 10 years preceding start of the pandemic (0, 1, >1), country of origin (4 categories), geographical region (5 groups), number of household members (0, 1, 2, 3+), probability of tobacco smoking (<10%, 10-<20%, 20+ %), bodymass index (< 25 kg/m^2^ / ≥ 25 kg/m^2^), family positive PCR swab test (at least one member of the family besides the index person with positive PCT test during the previous 2-3 weeks, yes/no) and Covid-19 vaccination (from date of second vaccination until end of follow-up).

c Occupations with fewer than 2000 employees kept as separate category within industrial sectors.

*Risk estimates across all industrial sectors*. Using the average JEM sum score to select industrial sectors and, within these, occupations of particular interest, there is a risk of leaving out occupations that score high on some COVID-JEM dimensions (for instance number of daily contacts and work with the general public), while scoring low on other dimensions (for instance job insecurity). For that reason and to provide a full systematic quantification of occupational risk independent of industrial sector and COVID-19 JEM assessment, we estimated the risk for all non-referent occupations with >2000 employees at the 4-digit DISCO-08 level while keeping occupations with ≤2000 and employees with missing DISCO-08 data as separate categories.

*Adjustments*. In all analyses, we first adjusted the risk estimate by sex and age, which are strongly related to both occupation and outcome (being a male and higher age conferring a higher risk). Secondly, we further adjusted for a fixed set of baseline variables according to the disjunctive confounder variable selection criteria (38). Included covariates are listed in the footnote to table 2 which also displays grouping categories. Social position, household income, number of children in the household and living in a large city were also considered as possible confounding factors but were not included in final statistical models because of strong overlap with other covariates within the same domain. Retirement, death, and emigration during follow-up were accounted for by censoring, while COVID-19 vaccination was controlled for by a time-varying variable.

All analyses were carried out in SAS 9.4 (SAS Institute, Cary, NC, USA).

## Results

**Figure 1:**
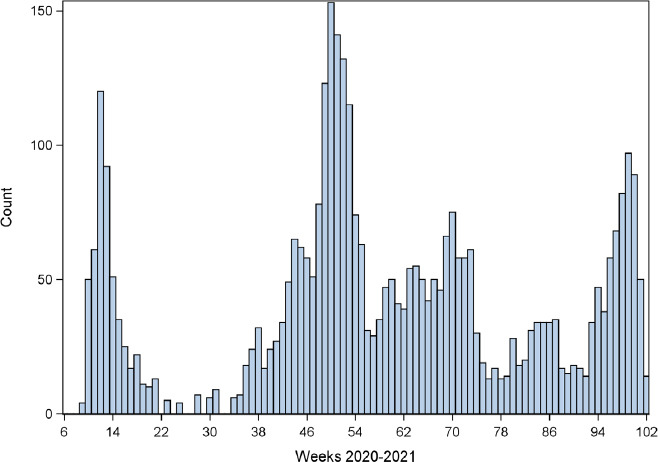
Weekly numbers of COVID-19 hospital admissions (total = 4 416) among employees (n = 2 451 542 in Denmark from week 8 of 2020 through week 50 of 2021 (data are omitted for weeks with fewer than 5 cases)

During 228.2 million person-weeks, we observed 4416 COVID-19 related hospital admissions peaking in weeks 12 and 50 of 2020, and week 47 of 2021 (figure 1, entire study population N=2 451 542). The corresponding numbers for the 155 occupations with >2000 employees and referents were 185.6. million person-weeks with 3 503 incident cases) The first vaccinations against COVID-19 took place in week 52 of 2020.

### Occupational risk by industrial sector

We identified six major industrial sectors defined by the 2-digit DB07 level with an average COVID-19 JEM sum score >12. Increased risk of COVID-19 related hospital admission was observed in one or more 4-digit DISCO-08 occupations within three of these (healthcare, social care and transportation), but not in education, retail sales and a heterogeneous group of service jobs) (table 2).

Within healthcare, eight of thirteen occupations with ≥2000 employees had an increased risk, with health care assistants experiencing the highest risk, and physiotherapists, midwives, dentists and dental assistants as notable exceptions (table 2).

In social care, four out of fourteen occupations had increased risk, the highest IRR being in nurses. Nursing aides (in institutions and private homes, respectively) and teachers/day care assistants for children aged 4–7 also had increased risk, while primary school teachers and social work and counselling professionals had not.

Among five jobs in the transportation sector, increased risk was seen among bus and possibly taxi drivers but not lorry and locomotive engine drivers.

### Occupational risk across all industrial sectors

Computation of IRR by 4-digit DISCO-08 occupations across all industrial sectors independently of COVID-19 JEM assessments revealed few additional occupations with elevated risk not captured by the industrial sector stratified analysis. These include construction managers, medical imaging and equipment operators, chefs, early childhood educators and food and related products machine operators (table 3 and supplementary material www.sjweh.fi/article/4063, table S1). Moreover, low risk was seen in seven occupations, mainly some high-skill and blue-collar occupations (supplementary table S1).

**Table 3:** Risk of Covid-19 related hospital admission for 155 non-referent 4-digit DISCO-08 occupations with > 2000 employees across all industrial sectors. Incidence rate ratios (IRR) with 95% confidence limits relative to employees in all occupations with unlikely occupational exposure to SARS-CoV-2 ^a^. The Table is continued in supplemental table 1.

Occupation (descending fully adjusted IRR)	DISCO-08 code	N employees	N Covid-19 admissions	IRR sex and age adjusted	IRR fully adjusted ^b^	95% CI
Construction Managers	1323	3 326	13	2.04	**2.22**	**1.28–3.85**
Medical Imaging and Equipment Operators	3211	2 646	8	2.25	**2.20**	**1.09–4.42**
Bus and Tram Drivers	8331	10 879	93	4.35	**2.13**	**1.64–2.77**
Chefs	3434	2 949	10	2.21	**2.02**	**1.07–3.84**
Generalist Medical Practitioners	2211	19 826	46	1.60	**1.93**	**1.28–2.91**
Psychologists	2634	8 072	15	1.43	**1.90**	**1.11–3.26**
Human Resource Managers	1212	2 884	9	1.87	1.90	0.98–3.68
Nursing Professionals	2221	63 600	134	1.64	**1.87**	**1.51–2.33**
Dairy Products Makers	7513	3 144	8	1.53	1.81	0.89–3.68
Social Work Associate Professionals	3412	7 019	23	2.14	**1.77**	**1.14–2.76**
Health Professionals Not Elsewhere Classified	2269	8 856	15	1.38	**1.77**	**1.02–3.05**
Healthcare Assistants	5321	44 641	134	2.18	**1.72**	**1.35–2.18**
Medical and Pathology Laboratory Technicians	3212	6 785	17	1.93	**1.68**	**1.00–2.83**
Packing, Bottling and Labelling Machine Operators	8183	2 166	8	2.18	1.68	0.83–3.39
Process Control Technicians Not Elsewhere Classified	3139	2 399	7	1.65	1.64	0.78–3.46
Software and Applications Developers and Analysts Not Elsewhere	2519	4 398	12	1.63	1.59	0.89–2.82
Medical Secretaries	3344	9 853	19	1.48	1.57	0.99–2.48
Receptionists (general)	4226	3 620	8	1.77	1.56	0.77–3.18
Journalists	2642	9 748	18	1.25	1.44	0.90–2.31
Home-based Personal Care Workers	5322	86 458	247	2.18	**1.43**	**1.16–1.78**
Food and Related Products Machine Operators	8160	17 038	57	1.98	**1.43**	**1.05–1.95**
Police Officers	5412	9 913	22	1.30	1.42	0.93–2.18
Physiotherapists	2264	9 615	13	1.05	1.38	0.78–2.46
Personal Services Workers Not Elsewhere Classified	5169	4 408	12	1.81	1.37	0.76–2.46
Musicians, Singers and Composers	2652	2 818	6	1.35	1.37	0.61–3.07
Locomotive Engine Drivers	8311	2 375	7	1.43	1.36	0.65–2.88
Special Needs Teachers	2352	3 895	7	1.16	1.35	0.60–3.04
Real Estate Agents and Property Managers	3334	3 134	6	1.31	1.35	0.60–3.02
Manufacturing Managers	1321	6 730	15	1.11	1.35	0.80–2.25
Contact Centre Information Clerks	4222	5 045	10	1.60	1.35	0.71–2.55
Early childhood educators	2343	70 233	133	1.42	**1.34**	**1.10–1.63**
Contact Centre Salespersons	5244	6 297	12	1.37	1.31	0.73–2.35
Electronics Engineering Technicians	3114	4 745	12	1.33	1.30	0.73–2.30
Building Frame and Related Trades Workers Not Elsewhere Classified	7119	3 309	10	1.67	1.29	0.68–2.45
Chemical and Physical Science Technicians	3111	9 663	22	1.57	1.28	0.83–1.96
Protective Services Workers Not Elsewhere Classified	5419	3 745	10	1.61	1.27	0.67–2.40
Hairdressers	5141	5 466	10	1.68	1.25	0.66–2.36
Missing 4-digit DISCO-08 code		337 306	693	1.35	1.11	0.81–1.53
Occupations with less than 2000 employees		124 287	220	1.07	1.08	0.91–1.28
Reference (all occupations with unlikely occupational SARS-CoV-2 exposure)1		369 341	559	1.00	1.00	

a Likelihood of occupational SARS-CoV-2 exposure according to a population-based international expert-rated job exposure matrix that assesses four measures of number of close indoor contacts at work, two mitigation measures and two job insecurity measures, each rated on a scale from low (0) to high (3),

b Adjustment for sex, age (10 year groups), duration of education at baseline (5 groups), number of hospital admissions for one or more of 11 chronic diseases in the 10 years preceding start of the pandemic (0, 1, >1), country of origin (4 categories), geographical region (5 groups), number of household members (0, 1, 2, 3+), probability of tobacco smoking (<10%, 10-<20%, 20+ %), bodymass index (< 25 kg/m^2^/ ≥ 25 kg/m^2^), family positive PCR swab test (at least one member of the family besides the index person with positive PCT test during the previous 2-3 weeks, yes/no) and Covid-19 vaccination (from date of second vaccination until end of follow-up). Significant associations in **bold.**

### Supplementary results

As expected, several demographic and social characteristics were unevenly distributed across categories of the COVID-19 JEM sum score, in particular, sex, age, duration of education and country of birth. Differences were less pronounced regarding size of household, health, and lifestyle (supplementary table S2).

Strong and robust sex- and age-adjusted associations were seen between COVID-19 related hospital admission and most covariates other than body mass index and positive PCR swab test of one or more family members in the previous 2–3 weeks (supplementary table S3)

## Discussion

In this nationwide registry-based follow-up study of COVID-19 related hospital admission during the first two years of the pandemic in Denmark, we observed increased risk in most occupations within healthcare, in several occupations within social care, and in a few occupations within transportation and various other occupations. Adjusted IRR in these at-risk occupations were generally in the range of 1.5–3. No increased risk was seen in the educational and retail sales sectors or in a number of service professions. Our analysis had the strength of being based on a large national cohort, but several potential sources of bias need consideration.

### Methodological issues

Hospital admission is not a perfect proxy for incidence of COVID-19. Admission rates among infected individuals are higher among those who are older, male, of non-white ethnicity, with various comorbidities, and have an unhealthy lifestyle (39, 40). To reduce any resultant bias, we adjusted risk estimates for age, sex, country of origin, history of hospital admission for other diseases, body mass index and tobacco smoking. Immunization, once it became available, will have reduced individual vulnerability to more severe infection requiring hospital admission, and therefore was a further factor of adjustment. There is also potential for confounding by non-occupational exposure to COVID-19. To reduce this bias, we adjusted for years of education, household size, and recent documentation of infection in a household member. A further complication is that during the study period, incidence of COVID-19 varied by region within Denmark, which could lead to bias where jobs were not uniformly distributed across the country. Therefore, we included adjustment for region. Despite these many adjustments, we cannot exclude some residual bias – for instance, we had no data on commuting to work by public transport.

A large number of comparisons inevitably produces chance associations, and real associations cannot be distinguished from spurious associations on statistical grounds. The main approach taken to address the multi-comparison problem was to focus on occupations that according to independent expert ratings a priori were more likely to confer increased risk of viral infection. Nevertheless, it should be acknowledged that demonstration of increased (or reduced) risk in some occupations could be the result of random variation regardless of statistical significance. Cautious interpretation and critical assessment of plausibility are needed before specific results are communicated or actions taken based upon findings related to specific occupations. The detail provided by stratification of jobs by industrial sector may be helpful in risk assessment because we expect that the same jobs in different sectors will in some cases have different risks. On the other hand, real risks in some occupations may have been missed because of small numbers. It was, for example, unexpected that the risk in midwives was not elevated, although an explanation might be that both pregnant women and midwives have been unusually careful to avoid infection.

Positive PCR swab tests among family members were only weak predictors of COVID-19 related hospital admission 2–3 weeks later despite an extremely high testing activity, particularly during the second year of the epidemic. This finding cannot be taken as strong evidence that viral transmission in the family is of minor importance. Since positive PCR tests are strongly clustered in families in time – many performed within hours the very same day – the sequence of positive testing in a family may not be a reliable indicator of the sequence of infection. Moreover, in spite of high test-activity, there may still be many undetected subclinical cases.

The follow-up period included the first two epidemic waves and the initial part of the third wave. The end of follow-up coincided with a time when the less virulent omicron virus variant outnumbered the more lethal alpha, beta, and delta variants. However, there is no reason to expect that this change will have affected occupations differentially, and therefore it should not have been a source of bias.

### Context and implications of findings

When comparing results across studies undertaken in different places and over different time periods, there is a possibility that differences in risk estimates could reflect not only the effects of bias and chance, but also variation in the extent of societal lockdown and effectiveness of precautionary measures as implemented in different countries at different stages of the pandemic. Results reported here may not apply to other countries or other time periods. Nevertheless, our findings are broadly consistent with those from elsewhere. They corroborate several earlier reports of increased risk among healthcare workers (1, 5–9, 12–14, 16, 21, 23–27), adding information on risk in various specific occupations not previously addressed in this sector – for instance medical laboratory technicians, hospital porters and psychologists. Increased risk among bus drivers has also been reported previously (18, 19). Elevated risk among shop assistants was observed in a mortality study (17) but not in our study. The absence of increased risk among teachers is notable, and again accords with other investigations (4, 41). That risk was increased in day care assistants providing daytime care for 4–7-year-old children might reflect closer contacts than experienced by primary school teachers, or that the latter were working from home when the epidemic peaked in Denmark in spring 2020 and winter 2021–22.

To the extent that the observed risk estimates reflect viral transmission related to working in a particular occupation, our data indicate that in the population and time period studied, recommendations and precautions to mitigate risk were insufficient and need to be revisited by employers and the Labour Inspection Agencies. When working at home is infeasible during periods with high community prevalence of infection, there is a need to develop and implement job- and task-specific guidelines based on the well-established general recommendations. At the same time, the demonstration of increased risk of infection in certain occupations may suggest interventions to protect the public more widely. For instance, elevation of risk among hairdressers might imply an increased risk for customers and family members too. As yet, however, there is little direct evidence of onward household transmission from people working in high-risk occupations (28, 42).

In most occupations where no or only marginally elevated risks (IRR <1.1) were observed, it seems likely either that viral transmission was not occurring in the workplace or that precautions were sufficient to mitigate it – as for instance in the travel, hotel and restaurant businesses, which have been locked down for long periods during the pandemic.

Significantly low risk estimates may have occurred in some jobs because limited contact with other people at work meant that SARS-CoV-2 transmission was lower than during leisure-time activities. Alternatively, they could be a consequence of random sampling variation in a context of multiple testing, or the reference groups used may not adequately have represented the lowest occupational exposure levels. The COVID-19 JEM was based upon subjective expert ratings and has so far not been validated (31).

The excessive societal impact of the COVID-19 pandemic may have consequences for occupational health reaching beyond the pandemic itself. In general, transmission of infectious diseases in the workplace has not been given high priority either in research agendas or in occupational health prevention programs (43, 44). This situation my change as documentation of the impact of workplace transmission of COVID-19 becomes stronger and widely accepted.

### Concluding remarks

Our study provides strong evidence that the workplace has been an important setting for viral transmission during the SARS-CoV-2 pandemic in Denmark, and that lock-down and other measures to reduce such transmission have not been fully effective. Ways should be sought to reduce risk further, especially in health and social care, and among bus and taxi drivers. In addition, there is mounting evidence to support compensation for COVID-19 as an occupational disease in a number of jobs.

## Supplementary material

Supplementary material
