# Re: Bonde JPE, Sell L, Flachs EM, Coggon D, Albin M, Hengel KMO, Kolstad H, Mehlum IS, Schlünssen V, Solovieva S, Torén K, Jakobsson K, Nielsen C, Nilsson K, Rylander L, Petersen KU, Tøttenborg SS. Occupational risk of COVID-19 related hospital admission in Denmark 2020–2021: a follow-up study. *Scand J Work Environ Health*. 2023;49(1):84–94. doi:10.5271/sjweh.4063

**DOI:** 10.5271/sjweh.4098

**Published:** 2023-05-01

After publication of this article, an error in the power calculation was discovered. The error can be found on page 87 of the original manuscript:

A power calculation based upon the outcome occurrence in the reference group indicated that a relative risk of **1.8** would be detectable in an occupation with 80% power at the 5% significance level if it included >2000 employees. **Therefore**, we report risk estimates for the non-referent occupations of this size or larger, while keeping all jobs regardless of size in the reference group.

The correct text should state:

A power calculation based upon the outcome occurrence in the reference group indicated that a relative risk of **approximately 3.2** would be detectable in an occupation with 80% power at the 5% significance level if it included >2000 employees. **Even confidence limits for occupations only including 2000 employees are broad**, we report risk estimates for the non-referent occupations of this size or larger, while keeping all jobs regardless of size in the reference group. Occupations with ≤2000 employees and employees with missing DISCO-08 codes were analyzed as separate categories.

