# Supplementary material for: Occupational risk of COVID-19 related hospital admission in Denmark 2020–2021: a follow-up study
Source: Scand J Work Environ Health. 2022 Dec 30;49(1):84–94. doi: 10.5271/sjweh.4063 (PMC10549918; doi:10.5271/sjweh.4063)
Supplement: Supplementary material [file SJWEH-49-84-S001.pdf]

Occupational risk of COVID-19 related hospital admission in Denmark 2020–2021: a follow-up study[1]

by Jens Peter Ellekilde Bonde, PhD,[2] Lea Sell, PhD, Esben Meulengracht Flachs, PhD, David Coggon, PhD, Maria Albin, PhD, Karen Marieke Oude Hengel, PhD, Henrik Kolstad, PhD, Ingrid Sivesind Mehlum, PhD, Vivi Schlünssen, PhD, Svetlana Solovieva, PhD, Kjell Torén, PhD, 14 Kristina Jakobsson, PhD, Christel Nielsen, PhD, Kerstin Nilsson, PhD, Lars Rylander, PhD, Kajsa Ugelvig Petersen, PhD, Sandra Søgaard Tøttenborg, PhD

1. Supplementary material
2. Correspondence to: Jens Peter Bonde, Department of Occupational and Environmental Medicine, Bispebjerg Frederiksberg Hospitals, Bispebjerg Bakke 23, DK-Copenhagen 2400 NV, Denmark. [E-mail: Jens.Peter.Ellekilde.Bonde@regionh.dk]

Table S1 (Table 3 continued). Risk of Covid-19 related hospital admission for 155 non-referent 4-digit DISCO-08 occupations with > 2000 employees across all industrial sectors. Incidence rate ratios (IRR) with 95% confidence limits relative to employees in all occupations with unlikely occupational exposure to SARS-CoV-2[1]

Occupation (descending adjusted IRR)	DISCO-08 code	N employees	N Covid-19 admissions	IRR sex and age adjusted	IRR fully adjusted[2]	95% CI	
Secretaries (general)	4120	7 280	13	1.31	1.22	0.70	2.12
Administrative and Executive Secretaries	3343	16 335	32	1.27	1.22	0.85	1.74
Childcare Workers	5311	48 101	92	1.50	1.21	0.97	1.52
Primary School Teachers	2341	76 740	118	1.08	1.21	0.99	1.48
Lawyers	2611	5 640	8	1.03	1.18	0.59	2.39
Managing Directors and Chief Executives	1120	21 681	48	1.10	1.18	0.88	1.59
Dentists	2261	3 441	6	1.21	1.18	0.52	2.67
Management and Organization Analysts	2421	8 561	15	1.16	1.17	0.70	1.95
Personal Care Workers in Health Services	5329	2 654	6	1.62	1.17	0.51	2.64
Armed Forces Occupations, Other Ranks	0310	9 046	11	0.83	1.16	0.59	2.28
Pharmacists	2262	4 701	8	1.21	1.14	0.54	2.42
Pharmaceutical Technicians and Assistants	3213	4 514	8	1.45	1.14	0.57	2.31
Mechanical Machinery Assemblers	8211	2 927	6	1.12	1.13	0.50	2.56
Social Work and Counselling Professionals	2635	15 861	23	1.16	1.12	0.73	1.71
Building caretakers	5153	18 843	51	1.31	1.11	0.81	1.53
Legal Professionals Not Elsewhere Classified	2619	16 063	23	1.07	1.11	0.72	1.69
Hand Packers	9321	7 336	18	1.63	1.08	0.67	1.75
Draughtspersons	3118	6 231	10	0.99	1.08	0.58	2.02
Cleaners and Helpers in Offices, Hotels and Other Establishments	9112	47 700	163	2.43	1.08	0.86	1.36
Special Teaching Professionals	2357	33 881	52	1.06	1.06	0.77	1.46
Electrical Mechanics and Fitters	7412	3 025	6	1.00	1.04	0.46	2.33
Civil Engineering Technicians	3112	11 501	19	0.93	1.02	0.65	1.62
Prison Guards	5413	2 742	5	1.07	1.01	0.41	2.45
Car, Taxi and Van Drivers	8322	6 282	21	1.84	1.01	0.64	1.59
Security Guards	5414	5 447	14	1.54	1.00	0.58	1.72
Other Cleaning Workers	9129	2 494	7	1.94	0.99	0.46	2.11
Financial and Investment Advisers	2412	18 636	27	0.90	0.97	0.66	1.44
Dental assistants and Therapists	3251	8 253	13	1.35	0.97	0.56	1.70
Systems Administrators	2522	4 084	7	0.96	0.97	0.46	2.04
Metal Processing Plant Operators	8121	5 859	11	1.02	0.94	0.51	1.73
Messengers, Package Deliverers and Luggage Porters	9621	3 487	9	1.67	0.93	0.48	1.81
Building and Related Electricians	7411	23 498	38	0.93	0.93	0.65	1.32
Plastic Products Machine Operators	8142	5 996	12	1.13	0.91	0.50	1.64
Civil Engineers	2142	13 422	20	0.87	0.91	0.58	1.42
Shop Sales Assistants	5223	67 961	87	1.04	0.90	0.70	1.17

Business Services and Administration Managers	1219	9 740	15	0.87	0.90	0.54	1.51
Retail and Wholesale Trade Managers	1420	7 284	10	0.89	0.90	0.48	1.70
Electronics Engineers	2152	3 709	5	0.73	0.89	0.36	2.24
Wood Processing Plant Operators	8172	3 853	6	0.84	0.89	0.40	2.01
Butchers, Fishmongers and Related Food Preparers	7511	3 688	5	0.91	0.89	0.36	2.17
Plumbers and Pipe Fitters	7126	12 712	22	0.96	0.88	0.56	1.38
Physical and Engineering Science Technicians	3119	7 893	11	0.79	0.88	0.48	1.60
Manufacturing Labourers Not Elsewhere Classified	9329	10 663	19	1.08	0.88	0.54	1.42
Kitchen Helpers	9412	13 907	27	1.49	0.88	0.58	1.33
Mail Carriers and Sorting Clerks	4412	8 302	17	1.15	0.88	0.53	1.45
Chemical Engineers	2145	5 178	7	0.87	0.87	0.39	1.91
Vocational Education Teachers	2320	10 245	14	0.78	0.85	0.50	1.46

Supplemental table S1 continued

Occupation (descending adjusted IRR)	DISCO-08 code	N employees	N Covid-19 admissions	IRR sex and age adjusted	IRR fully adjusted[2]	95% CI	
Cashiers and Ticket Clerks	5230	13 829	17	1.04	0.85	0.52	1.41
Education Managers	1345	4 772	6	0.69	0.83	0.37	1.87
Heavy Truck and Lorry Drivers	8332	23 114	39	0.86	0.82	0.58	1.17
Secondary Education Teachers	2330	15 128	15	0.65	0.82	0.49	1.38
Shelf Fillers	9334	31 167	54	1.04	0.81	0.60	1.11
Child Care Services Managers	1341	4 693	6	0.78	0.81	0.36	1.81
Painters and Related Workers	7131	9 332	14	0.97	0.79	0.45	1.36
Sales Workers Not Elsewhere Classified	5249	9 821	12	0.73	0.78	0.43	1.40
Blacksmiths, Hammersmiths and Forging Press Workers	7221	11 270	15	0.71	0.76	0.45	1.30
Information and Communications Technology User Support	3512	8 048	10	0.76	0.76	0.40	1.41
Stationary Plant and Machine Operators Not Elsewhere Classified	8189	6 962	11	0.87	0.75	0.41	1.39
Assemblers Not Elsewhere Classified	8219	7 062	9	0.73	0.74	0.38	1.45
Electrical and Electronic Equipment Assemblers	8212	4 086	6	0.87	0.72	0.32	1.63
Fast Food Preparers	9411	7 801	9	0.91	0.72	0.37	1.41
Welders and Flame Cutters	7212	5 134	7	0.72	0.70	0.33	1.49
Bricklayers and Related Workers	7112	10 252	13	0.73	0.68	0.39	1.21
Industrial and Production Engineers	2141	8 130	8	0.56	0.67	0.33	1.34
Motor Vehicle Mechanics and Repairers	7231	18 033	21	0.67	0.66	0.42	1.04
University and Higher Education Teachers	2310	29 501	27	0.63	**0.65**	**0.44**	**0.96**
Clearing and Forwarding Agents	3331	7 257	6	0.55	0.62	0.28	1.39
Civil Engineering Labourers	9312	24 198	31	0.67	**0.61**	**0.41**	**0.90**
Mechanical Engineers	2144	5 817	6	0.56	0.61	0.27	1.36
Software Developers	2512	25 789	26	0.60	**0.61**	**0.41**	**0.90**
Engineering Professionals Not Elsewhere Classified	2149	10 145	9	0.52	0.60	0.31	1.16
Lifting Truck Operators	8344	3 485	5	0.75	0.57	0.24	1.40
Gardeners Horticultural and Nursery Growers	6113	5 180	5	0.58	0.57	0.23	1.37
Building Construction Labourers	9313	11 123	14	0.70	**0.56**	**0.33**	**0.98**
Waiters	5131	10 921	9	0.73	0.56	0.28	1.09
Chemical Products Plant and Machine Operators	8131	4 368	6	0.76	0.53	0.24	1.20
Mechanical Engineering Technicians	3115	6 893	6	0.45	0.51	0.23	1.14
Metal Working Machine Tool Setters and Operators	7223	12 200	11	0.48	**0.49**	**0.26**	**0.90**
Cooks	5120	13 072	12	0.66	**0.47**	**0.26**	**0.84**
Carpenters and Joiners	7115	29 978	21	0.42	**0.46**	**0.30**	**0.71**

Missing 4-digit DISCO-08 code	-	337 306	693	1.35	1.11	0.81	1.53
Occupations with less than 2000 employees	-	124 287	220	1.07	1.08	0.91	1.28
Reference (all occupations with unlikely occupational SARS-CoV-2 exposure)[1]	-	369 341	559	1.00	1.00	-	-

1 Likelihood of occupational SARS-CoV-2 exposure according to a population-based international expert-rated job exposure matrix that assesses four measures of number of close indoor contacts at work, two mitigation measures and two job insecurity measures, each rated on a scale from low (0) to high (3), (31) This reference included 50 DISCO08 4-digit level jobs and constituted 15% of all employees (n = 365 738).

Table S2. Distribution of baseline characteristics of the study population grouped by Covid-19 JEM sumscores[1] (column percentages), n= 2 451 165(Employees deceased before start of follow-up (n=377) not included).

Characteristic (column percentages)		Low-level (JEM sumscore = 0) N = 369 341	Medium level (JEM sumscore 1-12 N = 579 256	Higher level (JEM sumscore >12-24) N = 1 165 262	Missing Covid-19 JEM value[2] N = 337 306
		%	%	%	%
Sex					
	Men	45.3	68.8	43.4	60.0
	Woman	54.7	31.2	56.6	40.0
Age, years					
	20 - <30	16.6	15.0	22.7	25.1
	30 - < 40	21.7	22.0	20.9	21.7
	40 - <50	26.1	25.8	22.4	22.7
	50 - <60	25.7	26.6	22.8	21.2
	60+	10.0	10.7	11.3	9.38
Geographical region					
	Capital	40.4	35.0	29.3	35.4
	Zealand	12.7	13.4	14.5	13.6
	South	15.0	17.7	19.9	18.1
	Central	22.8	22.8	23.5	22.1
	North	9.13	11.2	12.8	10.8
Duration of education, years					
	<= 10	28.0	29.9	28.9	22.4
	> 10 – 13	49.9	47.4	36.6	38.2
	> 13 – 16	18.1	16.3	26.3	26.9
	>16	2.43	5.00	6.50	8.13
	Missing	1.58	1.41	1.71	4.37
Country of birth					
	Denmark	90.2	90.7	85.6	79.0
	Other western countries	3.31	2.87	2.46	3.65
	Eastern Europe	2.81	2.68	4.49	8.75
	Other countries	3.71	3.74	7.50	8.61
Probability of tobacco smoking (JEM assigned)[3]					
	<10 %	4.12	5.65	3.97	1.26
	10 - <20 %	81.9	61.4	36.6	2.88
	20+ %	14.0	29.7	58.4	1.26
	Missing	0.00	3.31	1.12	94.6
Bodymass index kg/m^2 (JEM assigned)[3]					
	< 25	35.0	26.1	39.8	1.52
	≥25	65.0	70.6	59.1	3.88
	Missing	0.00	3.31	1.12	94.6
Number of hospital admissions 2010-2020[4]					
	0	83.8	85.0	82.8	86.4
	1	13.5	12.5	14.2	11.3
	≥2	2.72	2.55	3.03	2.38

**Supplemental table S2** continued					
Characteristic (column percentages)		Low-level (JEM sumscore = 0) N = 369 341	Medium level (JEM sum core 1-12 N = 579 256	Higher level (JEM sumscore >12-24) N = 1 165 262	Missing Covid-19 JEM value[2] N = 337 306
Number of household members					
	1	15.6	16.9	17.7	17.4
	2	34.6	33.0	34.2	33.1
	3	19.1	18.9	18.9	18.9
	4+	30.7	31.2	29.2	30.6
Number of seniors > 65 in household					
	0	95.6	96.1	95.1	95.8
	1+	4.36	3.95	4.87	4.22
Number of first-time positive PCR test (n=447 546) in households 2020-2021 (n = 1 596 543)					
	0	80.9	81.8	81.8	79.7
	1	11.8	11.1	11.1	12.3
	2+	7.40	7.10	7.10	8.03
Second Covid-19 vaccination obtained					
	1.01.2021 – 30.06.2021	66.4	65.6	61.6	55.0
	1.07.2021 – 14.12.2021	26.6	26.3	25.5	29.2

[1] Likelihood of occupational SARS-CoV-2 exposure according to a population-based international expert-rated job exposure matrix that assesses four measures of the number of close indoor contacts at work, two mitigation measures and two job insecurity measures, each rated on a scale from low (0) to high (3) (31).

[2] Missing Covid-19 JEM value because some DISCO-08 codes were available only at the 1-3 digit level, and some DISCO-88 code were missing. Two large groups of employees with specific codes in DISCO-08 but not in ISCO-88 were recoded to be included (day care workers (n=65 776) and special pedagogues (n=32 935), DISCO-08 codes 2343 and 2357, respectively).

[3] Sex-, age- and calendar period- specific JEM-values based upon Danish population surveys with more than 250 000 participants (36)

[4] Hospitalisation 2010-2020 for one or more of the following diseases (ICD-10): cancer (DC00-97), immune deficiency disease (DD80-81), diabetes mellitus (DE10-14), obesity (DE66), inflammatory nervous disease (DG00-09), neuromuscular disorder (DG66), cardiovascular disease (DI05-79), chronic lung disease (DJ40-79), chronic liver disease (DK71-75), inflammatory arthritis (DM0514), chronic kidney disease (DN00-19).

Table S3. Sex- and age-adjusted risk of Covid-19 related hospital admission 2020-21 by demographic, social and health characteristics at baseline. Incidence rate ratios (IRR) with 95% confidence intervals.

Characteristic		Number of Covid-19 hospital admissions	IRR sex and age adjusted	95% confidence intervals	
				Lower	Upper
Sex					
	Men	2 515	1.24	1.16	1.31
	Women (reference)	1 901	1.00	-	-
Age, years					
	20 - <30 (reference)	580	1.00	-	-
	30 - < 40	779	1.27	1.14	1.41
	40 - <50	1 002	1.46	1.32	1.62
	50 - <60	1 380	2.01	1.82	2.21
	60+	675	2.48	2.22	2.77
Geographical region					
	Capital	2 032	2.01	1.80	2.24
	Zealand	7991	1.75	1.55	1.98
	South	561	0.95	0.83	1.08
	Central	655	1.00	0.93	1.08
	North (reference)	369	1.00	-	-
Duration of education (years)					
	<= 10	369	1.54	1.36	1.34
	> 10 – 13	1 074	1.29	1.19	1.41
	> 13 – 16	1 783	1.00	0.93	1.08
	>16 (reference)	1 109	1.00	-	-
	Missing	81	1.24	0.99	1.56
Country of birth					
	Denmark (reference)	3 049	1.00	-	-
	Other western countries	106	1.13	0.93	1.37
	Eastern Europe	301	2.65	2.35	2.99
	Other countries	960	5.39	5.00	5.81
Probability of tobacco smoking[3] (JEM assigned)					
	<10 % (reference)	158	1.00	-	-
	10 - <20 %	1 750	0.96	0.82	1.14
	20+ %	1 800	1.25	1.06	1.47
	Missing	708	1.30	1.09	1.54
Bodymass index kg/m^2 (JEM assigned)[3]					
	< 25 (reference)	975	1.00	-	-
	≥25	2 733	1.08	0.98	1.19
	Missing	708	1.25	1.13	1.39
Number of hospital admissions 2010-2020[2]					
	0 (Reference)	3104	1	-	-
	1	927	1.79	1.67	1.93
	≥2	385	3.35	3.01	3.74

**Supplemental table S3** continued					
Characteristic		Number of Covid-19 hospital admissions	IRR sex and age adjusted	95% confidence limits	
Number of household members					
	1 (reference)	709	1	-	-
	2	1 468	0.97	0.89	1.06
	3	905	1.21	1.10	1.34
	4+	1 334	1.19	1.08	1.31
Completed vaccination against Covid-19					
	Yes	1 594	0.63	0.60	0.67
	No	2 822	1.00	-	-
Positive PCR test in family 2-3 weeks earlier (n = 98 739, 2.7% of all tests)					
	≥ 1	87	1.11	0.99	1.25
	0 (reference)	4329	1.00	-	-
Epidemic wave, weeks during follow-up					
	08 – 38 (reference)	748	1.00	-	-
	39 – 78	2 593	2.71	2.50	2.94
	78-102	1 075	1.88	1.71	2.06

[1] Sex-, age- and calendar period- specific JEM-values based upon Danish population surveys with more than 250 000 participants (36)

[2] Hospitalisation 2010-2020 for one or more of the following diseases (ICD-10): cancer (DC00-97), immune deficiency disease (DD80-81), diabetes mellitus (DE10-14), obesity (DE66), inflammatory nervous disease (DG00-09), neuromuscular disorder (DG66), cardiovascular disease (DI05-79), chronic lung disease (DJ40-79), chronic liver disease (DK71-75), inflammatory arthritis (DM0514), chronic kidney disease (DN00-19).
